# Ensemble refinement shows conformational flexibility in crystal structures of human complement factor D

**DOI:** 10.1107/S1399004713032549

**Published:** 2014-02-15

**Authors:** Federico Forneris, B. Tom Burnley, Piet Gros

**Affiliations:** a Crystal and Structural Chemistry, Bijvoet Center for Biomolecular Research, Department of Chemistry, Faculty of Science, Utrecht University, Padualaan 8, 3584 CH Utrecht, The Netherlands

**Keywords:** ensemble refinement, structural dynamics, complement system, proteolysis

## Abstract

Human factor D (FD) is a self-inhibited thrombin-like serine proteinase that is critical for amplification of the complement immune response. FD is activated by its substrate through interactions outside the active site. The substrate-binding, or ‘exosite’, region displays a well defined and rigid conformation in FD. In contrast, remarkable flexibility is observed in thrombin and related proteinases, in which Na^+^ and ligand binding is implied in allosteric regulation of enzymatic activity through protein dynamics. Here, ensemble refinement (ER) of FD and thrombin crystal structures is used to evaluate structure and dynamics simultaneously. A comparison with previously published NMR data for thrombin supports the ER analysis. The R202A FD variant has enhanced activity towards artificial peptides and simultaneously displays active and inactive conformations of the active site. ER revealed pronounced disorder in the exosite loops for this FD variant, reminiscent of thrombin in the absence of the stabilizing Na^+^ ion. These data indicate that FD exhibits conformational dynamics like thrombin, but unlike in thrombin a mechanism has evolved in FD that locks the unbound native state into an ordered inactive conformation *via* the self-inhibitory loop. Thus, ensemble refinement of X-ray crystal structures may represent an approach alternative to spectroscopy to explore protein dynamics in atomic detail.

## Abstract 2

Ensemble-refinement analysis of native and mutant factor D (FD) crystal structures indicates a dynamical transition in FD from a self-inhibited inactive conformation to a substrate-bound active conformation that is reminiscent of the allostery in thrombin. Comparison with previously observed dynamics in thrombin using NMR data supports the crystallographic ensembles.

## 1. Introduction   

Mammalian complement immune defence and blood coagulation depend on cascades of proteolytic reactions (Neurath & Walsh, 1976 ▶; Schenone *et al.*, 2004 ▶; Huntington, 2012 ▶; Ehrnthaller *et al.*, 2011 ▶). In these proteolytic cascades, regulatory mechanisms prevent unwanted proteolysis and transform the potentially broad and uncontrolled proteolysis into a finely regulated process (Krem & Di Cera, 2002 ▶). Regulation involves, amongst others, binding of ligands, cofactors or inhibitors (Adams & Huntington, 2006 ▶; Huntington, 2012 ▶), allostery (Di Cera *et al.*, 2007 ▶; Lechtenberg *et al.*, 2012 ▶) or enzyme degradation (Nilsson *et al.*, 2011 ▶). These mechanisms rely on precise protein structure and dynamics. In the complement system, the enzymatic activity of most proteases is tightly regulated through macromolecular interactions that ensure the effector functions: inflammatory responses, phagocytosis, lysis of pathogens and B-cell or T-cell stimulation (Dunkelberger & Song, 2010 ▶; Ricklin *et al.*, 2010 ▶). All complement proteases, except for factor D (FD), have regulatory domains located at the N-terminus. FD is a single-domain serine protease that has a unique physiological substrate. It converts the surface-bound alternative pathway pro-convertase C3bB into the active convertase C3bBb. This step is critical for rapid and localized amplification of the complement response.

FD belongs to the chymotrypsin family of serine proteinases. In contrast to most proteases of this family, human FD circulates in plasma in a mature but self-inhibited state (Narayana *et al.*, 1994 ▶; Supplementary Fig. S1*a* 1), resulting in a very low proteolytic reactivity towards peptide-substrate analogues (Taylor *et al.*, 1999 ▶). Binding of the physiological substrate C3bB involves extended surface-charge interactions and triggers conformational changes in FD, releasing the self-inhibitory lock and resulting in a catalytically active enzyme (Forneris *et al.*, 2010 ▶). The C3bB binding site, or ‘exosite’, in FD is composed of four surface loops and overlaps in part with the cofactor-binding regions and the Na^+^-binding loop critical for the proteolytic activity of the homologous thrombin and similar serine proteases (Supplementary Fig. S1). For thrombin, it has been shown that binding of Na^+^ and ligands to the surface loops stabilizes the enzyme and enhances activity, indicating a mechanism of allosteric regulation through affecting protein dynamics (Di Cera *et al.*, 2007 ▶; Huntington, 2008 ▶). However, crystal structures of wild-type FD show well defined and virtually identical conformations of the exosite loops in both unbound and substrate-bound states (Narayana *et al.*, 1994 ▶; Kim *et al.*, 1995*b* ▶; Jing *et al.*, 1998 ▶; Forneris *et al.*, 2010 ▶; Katschke *et al.*, 2012 ▶). Moreover, FD does not bind metal ions. Thus, allostery through protein dynamics appears not to play a role in activating FD. Nevertheless, mutations in FD in the region that corresponds to the Na^+^-binding site in thrombin enhance the enzymatic activity towards short peptides (Taylor *et al.*, 1999 ▶; Forneris *et al.*, 2010 ▶), and the structures of these mutants show increased disorder in surface loops reminiscent of the functional dynamics as observed in thrombin structures (Kim *et al.*, 1995*a* ▶; Forneris *et al.*, 2010 ▶).

Unbound wild-type FD has a structural motif in its catalytic site termed the self-inhibitory loop (residues 197–203, which correspond to residues 212–219 in chymotrypsin; throughout the text chymotrypsin numbering is given in parentheses), which is contiguous to loop 4 of the exosite (Supplementary Fig. S1). The self-inhibitory loop induces a nonproductive arrangement of the Asp–His–Ser catalytic triad and disrupts the proteolytic S1 and S2 sites (Narayana *et al.*, 1994 ▶). A salt bridge between Arg202 (Arg218) on the self-inhibitory loop and Asp177 (Asp189) locks the S1 pocket and rigidifies the exosite (Forneris *et al.*, 2010 ▶). In mammalian FDs (except for those from a few species, such as mouse and rat), the small hydrophilic Ser199 (Ser215) residue replaces a canonical tryptophan in the homologous proteases and is responsible for displacing the catalytic His41 (His57) from its canonical *gauche* conformation (Narayana *et al.*, 1994 ▶). A mutant of FD [triple mutant S81Y, T198S and S199W (S94Y, T214S and S215W)] designed to increase the structural similarity to trypsin had increased catalytic activity and its structure indicated an altered conformation of the self-inhibitory loop (Kim *et al.*, 1995*a* ▶). The structure of the ternary complex C3bBD [using FD mutant S183A (S195A) and determined at 3.5 Å resolution] shows the Asp–His–Ser catalytic site of FD in an enzymatically active conformation induced by substrate binding at the exosite (Forneris *et al.*, 2010 ▶). The structures of both the triple mutant (determined at 2.0 Å resolution; Kim *et al.*, 1995*a* ▶) and an R202A (R218A) mutant of FD (at 2.8 Å resolution; Forneris *et al.*, 2010 ▶) show active-site configurations that more resemble the active Asp–His–Ser triad of serine proteinases owing to rearrangements of the self-inhibitory loop. The mutation R202A (R218A) removes the Arg202–Asp177 (Arg218–Asp189) salt bridge (Forneris *et al.*, 2010 ▶), while steric hindrance of Trp199 (Trp215) in the triple mutant (Kim *et al.*, 1995*a* ▶) results in pronounced rearrangement of the proteinase active-site region. In these structures of free FD with an active-like conformation of the catalytic triad, the electron density consistently shows increased disorder in the surface-exposed loops, which is in contrast to the marked exosite rigidity observed in all previous FD crystal structures. These two FD variants have enhanced catalytic activities towards peptide substrates, but show reduced proteolysis rates towards the physiological C3bB substrate (Kim *et al.*, 1995*a* ▶; Forneris *et al.*, 2010 ▶).

The structural similarity to thrombin and differences in dynamical states prompted a further evaluation of both the structure and dynamics of FD. We determined a new structure of the FD R202A (R218A) mutant at higher resolution (1.8 Å) and subsequently applied ensemble refinement (ER; Burnley *et al.*, 2012 ▶) to model both structure and dynamics based on X-ray diffraction data sets for this structure and for a number of previously determined structures. A detailed analysis of the structure–function–dynamics relationship of thrombin based on NMR data is available (Lechtenberg *et al.*, 2010 ▶; Fuglestad *et al.*, 2012 ▶). We compared this analysis with the approach taken here by performing ER of several thrombin structures. ER indicated conformational dynamics in mutant FD structures reminiscent of thrombin allostery, suggesting that FD passes through a highly flexible state during its activation process.

## 2. Materials and methods   

### 2.1. Protein production, crystallization, data collection and refinement   

Human complement FD (UniProt entry P00746, residues 26–253) wild-type and mutant clones were generated as described previously (Forneris *et al.*, 2010 ▶) and subcloned into pUPE.05.05 expression vectors (U-Protein Express, Utrecht, The Netherlands) for tagless mammalian expression. The proteins were transiently expressed in HEK293-E cells (HEK293 cells that express EBNA1, supplied by U-Protein Express). Secreted FD was purified by cation exchange at pH 6.0 using a HiScreen Capto S column (GE Healthcare) followed by size-exclusion chromatography at pH 7.5 on a Superdex 75 column (GE Healthcare). For crystallization, FD R202A (R218A) was concentrated to 10 mg ml^−1^ and crystals were obtained by the hanging-drop vapour-diffusion technique at 18°C. The crystals grew after 6 d in a reservoir solution consisting of 15%(*w*/*v*) polyethylene glycol (PEG) 6000, 50 m*M* MES–NaOH pH 6.0. Diffraction data were collected on the X06SA beamline of the Swiss Light Source (SLS), Villigen, Switzerland. Data reduction was performed using *iMosflm* (Battye *et al.*, 2011 ▶) and *AIMLESS* (Evans, 2011 ▶); see Table 1 ▶ for statistics. The structure was solved using molecular replacement with *Phaser* (McCoy *et al.*, 2007 ▶) using the coordinates of wild-type FD (PDB entry 1dsu; Narayana *et al.*, 1994 ▶) as an initial search model. The model was improved by alternating cycles of manual model building using *Coot* (Emsley *et al.*, 2010 ▶) and restrained refinement using *PHENIX* (Adams *et al.*, 2010 ▶). Noncrystallographic symmetry and TLS were applied throughout the refinement. The final model was validated using *MolProbity* (Chen *et al.*, 2010 ▶) and deposited in the Protein Data Bank with accession code 4cbn. Structural figures were prepared using *PyMOL* (v.1.3r1; Schrödinger).

### 2.2. Ensemble refinement of FD and thrombin structures   

We performed ER for wild-type FD (Narayana *et al.*, 1994 ▶), the S183A mutant (Forneris *et al.*, 2010 ▶), the triple mutant S81Y/T198S/S199W (S94Y/T214S/S215W) (Kim *et al.*, 1995*a* ▶), the R202A mutant determined at 1.8 Å resolution and two crystal structures of FD bound to an antibody fragment specific for the FD exosite (Katschke *et al.*, 2012 ▶) (Table 1 ▶). For thrombin we analyzed Na^+^-bound thrombin (Figueiredo *et al.*, 2012 ▶; Huntington & Esmon, 2003 ▶), wild-type and mutant Na^+^-free thrombin (Johnson *et al.*, 2005 ▶; Carter *et al.*, 2004 ▶; Gandhi *et al.*, 2008 ▶) and thrombin–inhibitor complexes (Figueiredo *et al.*, 2012 ▶) for which structure factors were available and the resolution of the data was at least 2.0 Å (except for the E217K mutant of thrombin, the only available structure of which was determined at 2.5 Å resolution and which was included for completeness). A complete list of PDB files and associated literature references is given in Tables 2 ▶ and 3 ▶. Structural models were taken from the *PDB_REDO* server (Joosten *et al.*, 2012 ▶) and were first re-refined using the latest available version of *PHENIX* (Adams *et al.*, 2010 ▶). The structures from *phenix.refine* were subsequently used as input models for ER, which was performed as described previously (Burnley *et al.*, 2012 ▶; Burnley & Gros, 2013 ▶). In contrast to single-structure refinement, the data were modelled by an ensemble of structures obtained by maximum-likelihood time-averaged restrained MD simulation. The averaging window during the simulation was determined by τ_*x*_, which is by default derived from the resolution of the diffraction data. The contribution of lattice disorder was modelled by an overall TLS model deduced from the *B* factors of the dynamically stable core of the molecule (Burnley *et al.*, 2012 ▶). This results in an overall TLS model (typically with *B*_TLS_ < *B*_refined_) that is applied to the whole molecule as an underlying *B*-factor model present during the sampling of the structures. In addition to this overall TLS model, further disorder was modelled by atomic fluctuations as observed in the trajectory of structures obtained during the simulation. Finally, the reported number of structures describing the ensemble was reduced by selecting the minimal number of structures, equally distributed over the sampling time, needed to reproduce the *R* factor to within 0.1%. In the figures 25 models per ensemble are shown for clarity.

### 2.3. Biophysics   

For the biophysical analyses, we considered wild-type FD, the R202A mutant and an additional variant of FD bearing a surface mutation distant from both the exosite and the catalytic site [R106A (R121A)], which was used as a control. All samples were dialyzed in 50 m*M* sodium phosphate pH 8.0 with 150 m*M* NaCl before analysis.

Circular-dichroism measurements were carried out on a Jasco 600 spectropolarimeter using FD samples at a concentration of 1–2 mg ml^−1^ in a 0.2 mm path-length cell, with a 1 nm bandwidth, 0.1 nm resolution, 1 s response time and a scan speed of 20 nm min^−1^.

For Thermofluor analysis (Pantoliano *et al.*, 2001 ▶), a volume of 25 µl of a solution containing 250 ng protein was diluted in H_2_O with 5× SYPRO Orange (Sigma–Aldrich) and the increase in fluorescence was monitored on a MyiQ real-time PCR instrument (Bio-Rad). All conditions were assessed in triplicate. Assays were performed over the temperature range 15–90°C using a ramp rate of 1°C min^−1 ^in steps of 0.5°C. The apparent *T*_m_ was determined as the inflection point of a sigmoidal fit to the normalized fluorescence signal.

Analysis of thermal denaturation parameters using intrinsic protein fluorescence was performed using FD samples at 1 mg ml^−1^. Assays were performed using an Optim 100 reader (Avacta Analytics) over the temperature range 20–90°C using a ramp rate of 1.0°C min^−1^ in steps of 1.0°C.

## 3. Results and discussion   

### 3.1. Structure of the FD R202A mutant at 1.8 Å resolution   

**Figure 1:**
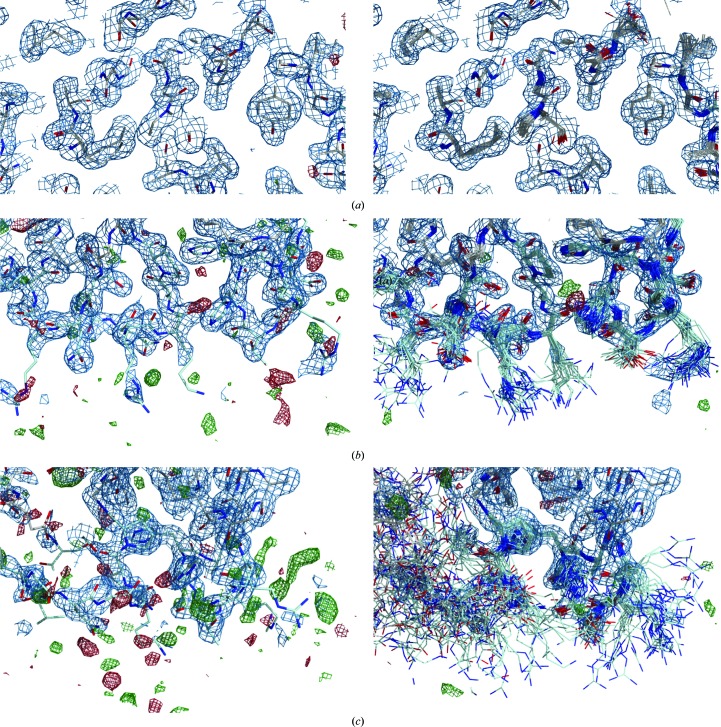
Comparison of electron densities for the FD R202A (R218A) structure at 1.8 Å resolution refined using conventional refinement (left) and ensemble refinement (right). Shown are (*a*) an ordered region in the core of the FD molecule, (*b*) the exosite region for chain *B* of the asymmetric unit, showing disordered side chains for the surface-exposed residues, (*c*) the exosite region for chain *A* of the asymmetric unit, showing poorly defined electron density for the main chain resulting in large displacements of the ensemble models. The panels show the 2*mF*_o_ − *DF*_m_ map (blue) contoured at 0.38 Å e^−3^ (1.00σ) and the *mF*_o_ − *DF*_m_ map (green and red) contoured at ±0.31 Å e^−3^ (±2.45σ for standard refinement, ±3.01σ for ER).

The previously reported crystal structure of FD R202A determined at 2.8 Å resolution (Forneris *et al.*, 2010 ▶) showed the catalytic site in an active configuration with residual density indicating the presence of disorder, which could not be modelled. In addition, these data yielded weak electron density for a number of surface-exposed loops, suggesting high mobility and/or multiple conformations for these regions (Fig. 1 ▶). New crystals of the FD R202A (R218A) mutant were obtained and a data set was collected to high resolution (1.8 Å; see Table 1 ▶ for data-collection and refinement statistics). The new map allowed a more detailed interpretation of the active site, showing multiple conformations that indicate the coexistence of both active and inactive configurations (Supplementary Fig. S2). Despite the overall improvement in electron density, however, the surface-exposed regions corresponding to the exosite loops remained poorly defined, with only few interpretable side chains (Fig. 1 ▶, Supplementary Fig. S3).

### 3.2. Ensemble refinement of the FD crystal structures   

**Figure 2:**
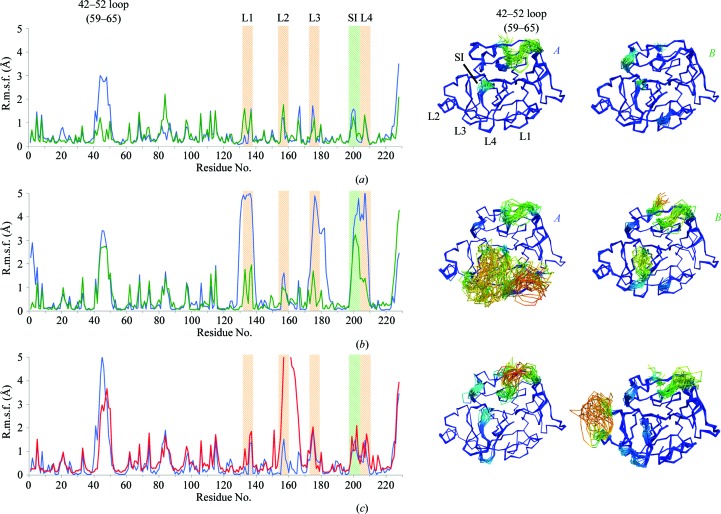
ER of FD crystal structures. Ensemble structures are coloured by r.m.s.f., ranging from 0 to 5 Å; the r.m.s.f. was calculated per residue for all non-H atoms. (*a*) Wild-type FD (original PDB entry 1dsu), with chains *A* (left) and *B* (right) plotted in blue and green, respectively; (*b*) R202A (R218A) FD mutant (original PDB entry 4cbn), with chains *A* (left) and *B* (right) plotted in blue and green, respectively; (*c*) S183A (S195A) (original PDB entry 2xw9) and the triple mutant of FD (original PDB entry 1dst) shown on the left and right and plotted in blue and red, respectively. Coloured areas indicate the location of the 42–52 (59–65) flexible loop, the four exosite loops, spanning residues 132–135 (145–149) (L1), 155–159 (161–169) (L2), 173–176 (185–188) (L3) and 203–209 (219–224) (L4), and the self-inhibitory loop (SI). Models are coloured by r.m.s.f. from blue through green to red.

Application of ER to the data set for FD R202A (R218A) collected at 1.8 Å resolution significantly reduced the *mF*_o_ − *DF*_m_ electron-density differences (Fig. 1 ▶, Supplementary Fig. S3), with a small improvement of 0.6 percentage points in *R*_free_ (Table 2 ▶). ER modelled a large number of alternative conformations for the exosite loops, with root-mean-square (r.m.s.) differences in amino-acid positions of up to 6 Å (Fig. 2 ▶). Different crystal-packing environments of the two copies in the asymmetric unit showed different amounts of disorder of the exosite loops, as indicated by large displacements of the exosite loops 1, 3 and 4 in the first copy and of loop 4 in the other copy (Fig. 2 ▶). We initially observed an unfolding of the N-terminus that did not account for the observed electron density; therefore, for subsequent runs of this data set a weak harmonic restraint was placed on the C^α^ atom of the terminal Ile. To assert the consistency of the ensemble model, ten parallel simulations were performed differing only in the random number seed used to generate the initial velocities (Supplementary Fig. S4). The ensemble repeats show high real-space correlation values between ensemble repeats, the majority of which are greater than 0.95, with a minimum of 0.60 around the exosite loops showing high flexibility, demonstrating the reproducibility of the ER method.

ER of the wild-type, S183A (S195A) and S81Y/T198S/S199W (S94Y/T214S/S215W) FD data sets showed improvements in *R*_free_ of 1.4–2.4% compared with single-structure models (Table 2 ▶). The resulting ensembles yielded atomic root-mean-square fluctuation (r.m.s.f.) values that showed similar distributions over the protein chain (Fig. 2 ▶). Consistent with previous reports (Narayana *et al.*, 1994 ▶; Kim *et al.*, 1995*a* ▶; Jing *et al.*, 1998 ▶; Forneris *et al.*, 2010 ▶), a pronounced disorder around residues 42–52 (59–65) was observed in all ensembles for these FD data sets. Notably, the wild-type and S183A (S195A) FD data sets showed well defined single conformations for the self-inhibitory and exosite regions, whereas the triple mutant FD displayed high mobility similar to the R202A (R218A) mutant. However, this flexibility was restricted to exosite loop 2 (Fig. 2 ▶). Thus, the ensemble models reflect that FD adopts an overall stable structure, except for those with mutations [R202A (R218A) and S81Y/T198S/S199W (S94Y/T214S/S215W)] in the self-inhibitory loop, in which marked flexibility is observed in the exosite loops. Biophysical analyses using thermal shift assays and circular-dichroism spectroscopy showed very similar results for wild-type and R202A (R218A) FD (Supplementary Fig. S5). Thus, the highly flexible R202A (R218A) variant is properly folded in solution at room temperature and its thermal stability is the same as that observed for wild-type FD, with a variation in the *T*_m_ values of less than 1°C between the wild type and the R202A (R218A) mutant.

**Figure 3:**
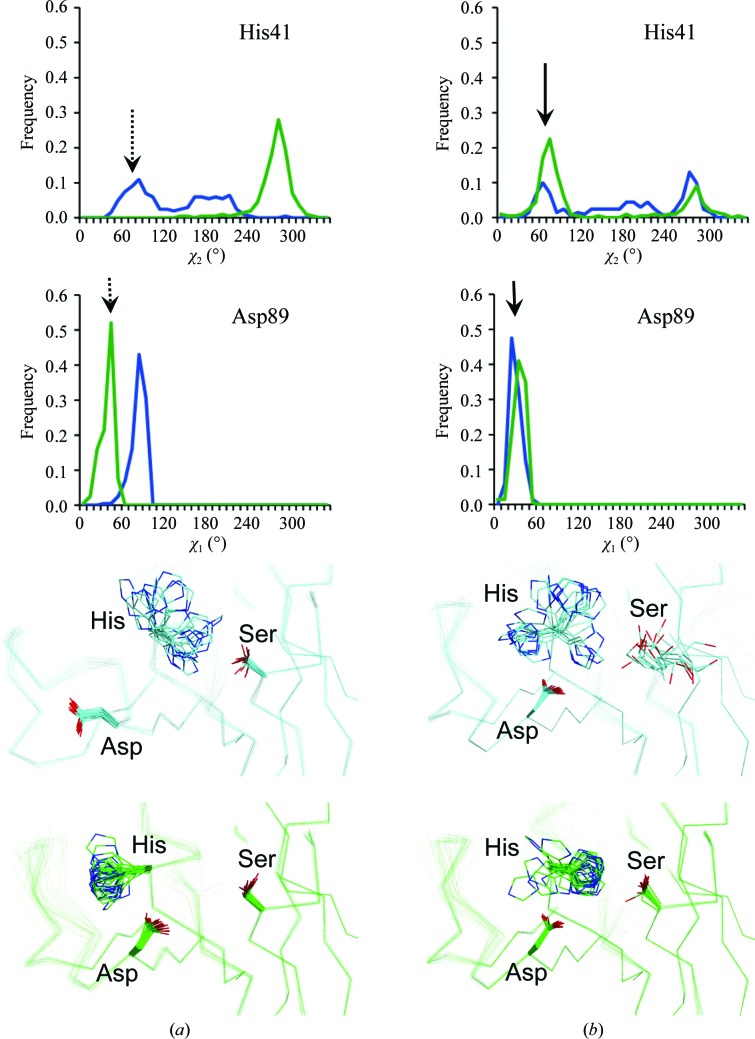
χ_2_ and χ_1_ distributions of the catalytic His41 and Asp89 residues, respectively, of FD observed in the ensemble structures of (*a*) wild-type FD (original PDB entry 1dsu) and (*b*) the R202A (R218A) mutant (original PDB entry 4cbn). The arrows indicate the angular value compatible with the active conformation of the serine protease catalytic site. The dashed arrow for wild-type FD indicates that there are no active conformations of His41 (His57) and Asp89 (Asp102) at the same time in the same copy. Both structures contain two copies in the asymmetric unit; data are shown in blue and green for chains *A* and *B*, respectively.

The ensembles of the various FD data sets revealed different dynamic distributions of active and inactive conformations of the Asp–His–Ser catalytic triad (Fig. 3 ▶). For both copies in the asymmetric unit of wild-type FD, ER confirmed the catalytically inactive arrangements observed in the original structure of wild-type FD (Fig. 3 ▶ *a*), with either Asp89 (Asp102) or His41 (His57) pointing out of the catalytic site (Narayana *et al.*, 1994 ▶). While Ser199 (Ser215) of the self-inhibitory loop blocks the formation of the canonical active configurations, the two copies in the asymmetric unit respond differently: either His41 (His57) adopts the inactive (but more stable) *trans* conformation or loop 79–90 (92–103) rearranges to allow Asp89 (Asp102) to flip out. The arrangement of the catalytic site of FD R202A was originally reported with a 100% active conformation in both copies of the 2.8 Å resolution crystal structure (Forneris *et al.*, 2010 ▶). For the 1.8 Å resolution data set of this mutant, the ensemble resulted in distributions of 30%:70% and 80%:20% active:inactive conformations for the two copies in the asymmetric unit (Fig. 3 ▶ *b*). Partially active conformations of the catalytic site in FD R202A (R218A) *versus* completely inactive conformations for the wild type are consistent with the observed increase in the catalytic activity of FD R202A (R218A) towards artificial peptide substrates. ER of the S81Y/T198S/S199W (S94Y/T214S/S215W) triple mutant of FD highlighted the large conformational distortions in the FD structure owing to the three mutations introduced in proximity to the catalytic site, the self-inhibitory loop and the S1 pocket. In agreement with the original observations by Kim *et al.* (1995*a* ▶), this ensemble showed a modified but still rigid conformation of the self-inhibitory loop, with Asp177 (Asp189) forming a salt bridge to Arg207 (Arg223). This rearrangement results in a 100% active conformation of the catalytic site and enhanced catalytic activity towards peptide substrates. A distribution of active and inactive conformations of the catalytic residues of FD was also observed in the crystal structure of human FD bound to a specific antibody fragment (Katschke *et al.*, 2012 ▶), showing a multi-rotamer distribution for the catalytic residue His41 that is remarkably similar to that observed in the R202A (R218A) FD mutant ensemble. The antibody binds to a region of the FD exosite, thereby creating steric hindrance for binding of the C3bB substrate. In this FD–antibody structure, the Arg202–Asp177 (Arg218–Asp189) self-inhibitory lock is retained. However, antibody-bound FD displayed enhanced proteolytic activity towards artificial peptide substrates compared with the wild type, similar to the FD R202A (R218A) mutant. In this case, ER showed conformational flexibility only at the active site, providing an explanation of the antibody-induced increase in catalytic activity (Supplementary Fig. S6).

### 3.3. Comparison of ER and NMR results for thrombin   

**Figure 4:**
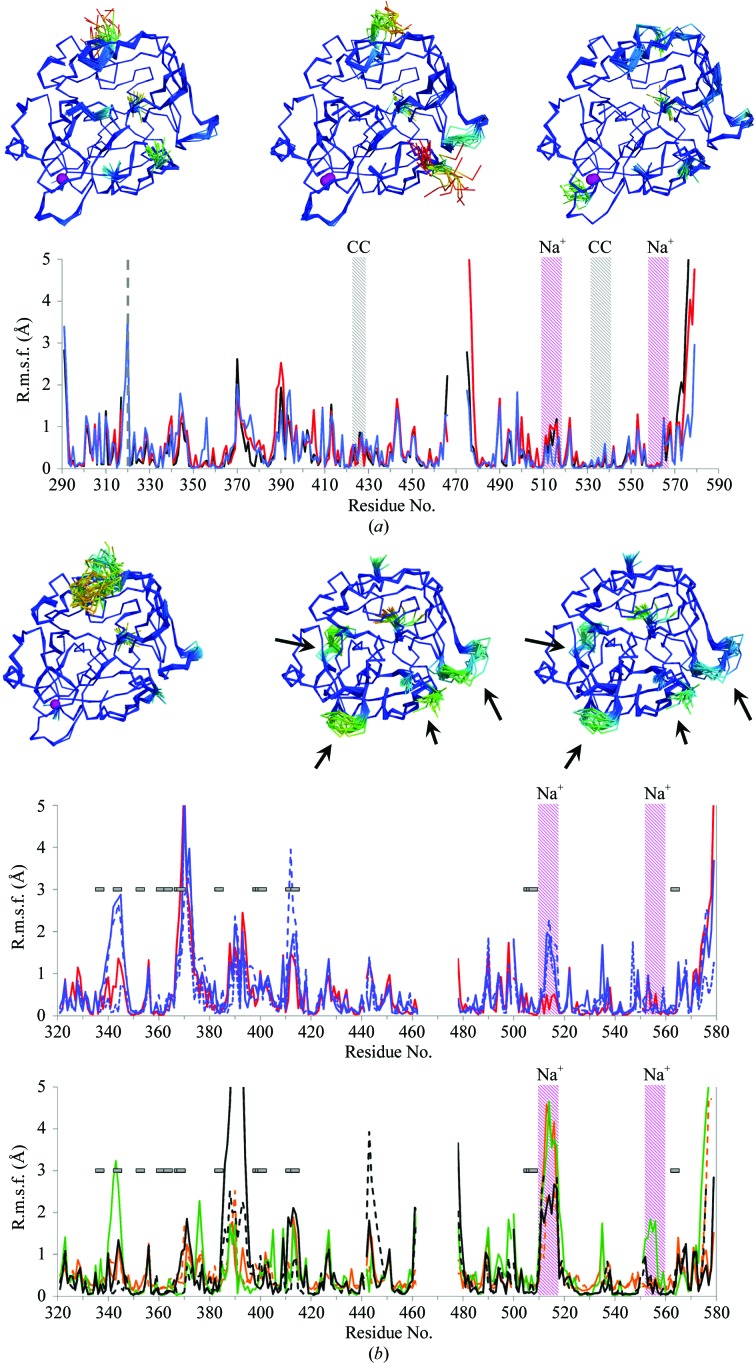
ER of thrombin crystal structures. (*a*) Thrombin ensembles obtained from ER of data sets of thrombin in complex with PPACK derivatives (Figueiredo *et al.*, 2012 ▶): d-Phe-Pro-d-Arg-Cys-CONH_2_ (original PDB entry 3u8t, black), d-Phe-Pro-d-Arg-Ile-CONH_2_ (original PDB entry 3u8r, red) and d-Phe-Pro-d-Arg-Thr-CONH_2_ (original PDB entry 3u8o, blue). Models are coloured by r.m.s.f. from blue through green to red. The bound sodium ions are shown as purple spheres. In the r.m.s.f. plots, the grey dashed line indicates the separation between thrombin light and heavy chains. Coloured areas indicate the sodium-binding loops (Na^+^, purple) and the regions involved in crystal-packing contacts (CC, grey). (*b*) Ensembles and r.m.s.f. plots for wild-type Na^+^-bound thrombin (original PDB entry 3u69) and the two monomers observed in the asymmetric unit of wild-type Na^+^-free thrombin (original PDB entry 2afq). Models are coloured by r.m.s.f. from blue through green to red. The bound sodium ions are shown as purple spheres. The histograms show Na^+^-bound wild-type thrombin (original PDB entry 3u69, red) and the S183A mutant (original PDB entry 1jou, blue) (top) and Na^+^-bound wild-type thrombin (original PDB entry 2afq, black), the E217K mutant (original PDB entry 1rd3, orange) and the D102N mutant (original PDB entry 3bei, green) (bottom). Purple areas indicate the Na^+^-binding loops. For structures with multiple copies in the asymmetric unit, solid lines are shown for chains *A* and *B*, dashed lines for chains *C* and *D*, and dotted lines for chains *E* and *F*. Arrows and horizontal bars indicate regions with observed significant chemical shift differences (≥0.1 p.p.m.) during the transition from Na^+^-bound to Na^+^-free thrombin according to Lechtenberg *et al.* (2010 ▶).

Quantitative, direct measurements of protein dynamics of thrombin by solution NMR provide an orthogonal benchmark for the fluctuations observed in the X-ray ensemble models. Fuglestad *et al.* (2012 ▶) performed high-quality NMR relaxation experiments on thrombin in complex with the inhibitor PPACK. Figueiredo *et al.* (2012 ▶) published high-resolution X-­ray crystal structures of a series of three PPACK derivatives. We re-refined these structures using ER (refinement statistics and a comparison with the published data are summarized in Table 3 ▶). Overall, the *R*_free_ improved by 0.3–1.9 percentage points. Fig. 4 ▶(*a*) displays the atomic r.m.s.f. for these ensembles. These r.m.s.f. values indicate regions exhibiting elevated dynamics similar to those deduced from the *R*_ex_ and *S* ^2^ values as depicted in Figs. 3(*b*) and 3(*d*), respectively, of Fuglestad *et al.* (2012 ▶). For loops 424–428 (107–110) and 531–540 (201–208), however, the r.m.s.f. values observed in the ensemble are significantly smaller, which may be attributed to the local effects of crystal-packing interactions (Fig. 4 ▶ *a*). The γ loop, the N- and C-termini of the light chain and the C-terminus of the heavy chain all exhibit high r.m.s.f. values in the ensemble structures in agreement with high *R*_ex_ rates (from motions on the micosecond-to-millisecond time scale) and low *S* ^2^ parameters (sensitive to sub-nanosecond dynamics), supporting the observation of molecular disorder in these regions. The 30s, 60s and 70s loops show high r.m.s.f. in the ensemble structures; however, these regions were not quantified in the NMR experiments because these residues could not be assigned. The lack of assignment is most likely to be caused by peak broadening owing to conformational exchange, indicative of large motions at these positions. Thus for the most part, ER samples protein dynamics in crystal structures that concur with observations made by NMR spectroscopy in solution.

Similarly, the application of ER to selected Na^+^-bound and Na^+^-­free data sets available for thrombin (Fig. 4 ▶ *b*) shows areas of high r.m.s.f. that coincide with pronounced changes in chemical shift (Lechtenberg *et al.*, 2010 ▶) and supports the enhanced exosite flexibility of this proteolytic enzyme in the absence of its ligands. In addition, the r.m.s.f. values obtained from ER of these data sets are in agreement with a comprehensive evaluation of r.m.s. deviations for C^α^ positions of Na^+^-­bound and free thrombin structures (Huntington, 2008 ▶). Previously, ER was successful in highlighting functional protein dynamics captured in crystal structures, as shown by the comparison of NMR and crystallographic data for proline isomerase (Burnley *et al.*, 2012 ▶). These new data on thrombin further support the reliability of ER for the evaluation of protein flexibility using X-ray diffraction data.

## 4. Conclusions   

Many serine proteinases show allosteric regulation through highly flexible intermediate states (Hauske *et al.*, 2008 ▶; Merdanovic *et al.*, 2013 ▶; Huntington, 2008 ▶; Di Cera *et al.*, 2007 ▶). These intermediates are stabilized by ligand binding at multiple exosites located on their surfaces. Based on the available biochemical and structural data, complement FD has been classified as an unusual serine proteinase owing to its self-inhibitory mechanism and its marked rigidity. Using ER, we detected a highly flexible state of an FD variant [R202A (R218A)]. The R202A (R218A) mutation disrupts a crucial interaction responsible for maintaining the self-inhibited state of FD. Release of this conformational interlock results in increased catalytic activity towards artificial peptides and displays a distribution of inactive and active conformations of the catalytic site (60%:40% active:inactive conformations of the catalytic triad, in contrast to the 100% inactive conformation observed in wild-type FD). The various FD data sets also showed distributions of multiple conformations of the active-site residues, which were affected in detail by the crystal-lattice environment. As shown previously by Narayana *et al.* (1994 ▶), the wild-type FD presents a striking example of this, in which the two copies in the asymmetric unit show conformations that are completely inactive (in agreement with the biological data) but that are inactive in two totally distinct ways, with either the catalytic Asp or His flipped out. These discrepancies arising from the crystal-packing environment clearly affect the interpretation of protein dynamics from crystallographic data. In the case of FD, the ensembles indicate that the active site is dynamic, reflecting the concerted conformational rearrangements for residues located in the catalytic site, the self-inhibitory loop and the exosite.

In thrombin, allosteric transitions from Na^+^-free to Na^+^-bound states affect protein stability and enzymatic activity. ER of the FD R202A mutant highlights increased exosite dynamics upon release of the Arg202–Asp177 (Arg218–Asp189) self-inhibitory lock, revealing a highly flexible state reminiscent of an ensemble of conformational states traditionally described as the Na^+^-free ‘slow’ form of thrombin (Adams & Huntington, 2006 ▶). The dual role of Arg202 (Arg218) in FD self-inhibition and substrate recognition (Forneris *et al.*, 2010 ▶) suggests that the conformational dynamics of the FD R202A (R218A) mutant ensemble may represent a highly flexible intermediate state between the disruption of the Arg202–Asp177 (Arg218–Asp189) self-inhibitory lock and interaction with binding partners. Thus, during activation FD may pass through an intermediate step regulated by protein dynamics, similar to thrombin allostery. Under physiological conditions FD is always stabilized by intramolecular or intermolecular interactions that avoid this flexible intermediate. In the free state of the enzyme, the exosite of FD shows a rigid conformation owing to self-inhibitory intramolecular interactions, whereas upon substrate recognition the exosite flexibility is prevented by extended intermolecular interactions with the substrate. Taken together, our data suggest that FD indeed exhibits conformational dynamics similar to thrombin and other serine proteinases, but that unlike thrombin a mechanism has evolved in mammalian FD that locks the unbound native state into an ordered conformation *via* the self-inhibitory loop.

Modelling X-ray diffraction data using ER provides detailed information about protein dynamics, enhancing the information content of the structural models derived from experimental diffraction data (Burnley *et al.*, 2012 ▶). Our analysis further supports the relevance of the distributions obtained by ER of the crystallographic data. Thus, for many cases such as FD, where complete solution NMR analysis is unachievable, ER of protein-crystal diffraction data sets provides an alternative method to explore the dynamics of protein structures in atomic detail. Given the large amount of crystal structures solved and available for analysis, structural refinement using ER may thus reveal hitherto unmined details about protein dynamics. The challenge, however, will be to link the observed detailed protein structure and dynamics to the biological function of the molecules.

## Supplementary Material

PDB reference: complement factor D mutant R202A after conventional refinement, 4cbn

PDB reference: complement factor D mutant R202A after ensemble refinement, 4cbo

Supporting Information.. DOI: 10.1107/S1399004713032549/rr5061sup1.pdf

## Figures and Tables

**Table 1:** Data-collection and refinement statistics for FD R202A Values in parentheses are for the highest resolution shell.

Data collection	
Beamline	X06SA, SLS
Space group	*P*2_1_2_1_2_1_
Unit-cell parameters	
*a* (Å)	44.14
*b* (Å)	67.49
*c* (Å)	133.59
Resolution (Å)	47.33–1.80 (1.84–1.80)
*R*_merge_ † (%)	14.1 (51.2)
No. of unique reflections	37211 (2186)
Completeness (%)	99.0 (99.1)
Multiplicity	3.4 (3.3)
〈*I*/σ(*I*)〉	4.9 (1.9)
CC_1/2_ ‡	0.964 (0.615)
Refinement	
Protein atoms	3604
Water molecules	308
*R*_work_/*R*_free_ § (%)	18.98/21.87
Average *B* value for all atoms (Å^2^)	29.20
R.m.s. deviations from ideal values	
Bond lengths (Å)	0.007
Bond angles (°)	1.21
Ramachandran statistics (%)	
Favoured	95.4
Allowed	3.8
Outliers	0.8

† *R*_merge_ = 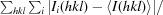 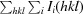, where *I_i_*(*hkl*) is the observed intensity for a reflection and 〈*I*(*hkl*)〉 is the average intensity obtained from multiple observations of symmetry-related reflections.

‡ CC_1/2_ is the mean(*I*) correlation between half sets, as defined by Karplus & Diederichs (2012 ▶).

§ *R*_work_ and *R*_free_ are crystallographic *R* factors calculated for the working and test data sets (Brünger, 1992 ▶).

**Table 2:** Re-refinement and ensemble-refinement statistics for FD data sets

	Wild type	R202A	S183A	Triple mutant	Antibody-bound (form 1)	Antibody-bound (form 2)
PDB code†	1dsu	4cbn	2xw9	1dst	4d9r	4d9q
Resolution (Å)	2.0	1.8	1.2	2.0	2.4	2.3
*phenix.refine* re-refinement						
*R*_work_ ‡	0.149 (0.188)	0.190 (N/A)	0.156 (0.145)	0.168 (0.193)	0.194 (0.204)	0.192 (0.184)
*R*_free_ ‡	0.188 (0.203)	0.219 (N/A)	0.172 (0.176)	0.200 (0.213)	0.231 (0.245)	0.220 (0.223)
Geometric r.m.s.d.						
Bonds (Å)	0.004	0.007	0.008	0.004	0.004	0.004
Angles (°)	0.82	1.24	1.33	0.96	0.87	0.85
Dihedrals (°)	12.8	15.4	13.3	12.2	14.2	12.9
Ramachandran statistics (%)						
Outliers	0.2	1.3	0.4	0.9	0.2	0.0
Allowed	3.4	3.6	2.0	3.2	4.1	3.1
Favoured	96.3	95.2	97.6	95.9	95.7	96.9
*phenix.ensemble_refinement*						
τ_*x*_ (ps)	0.5	0.6	1.4	0.5	1.0§	1.0§
No. of models	50	77	170	84	800	800
*R*_work_	0.117	0.166	0.125	0.142	0.183	0.182
*R*_free_	0.164	0.213	0.152	0.186	0.239	0.219
Geometric r.m.s.d. (centroid distribution)						
Bonds (Å)	0.005	0.009	0.008	0.004	0.006	0.006
Angles (°)	0.87	1.12	1.15	0.75	1.04	0.931
Dihedrals (°)	8.0	8.6	7.6	7.0	9.2	9.14
Geometric r.m.s.d. (whole distribution)						
Bonds (Å)	0.008	0.014	0.015	0.008	0.009	0.006
Angles (°)	1.27	1.78	1.95	1.23	1.08	1.05
Dihedrals (°)	16.7	17.6	16.9	16.7	16.2	15.6
Ramachandran statistics (whole distribution) (%)						
Outliers	1.4	5.6	2.7	3.4	4.8	3.6
Allowed	6.0	8.6	5.7	8.6	10.3	8.3
Favoured	92.7	85.8	91.7	88.0	84.9	88.1
Ramachandran statistics (per torsion angle) (%)						
Outliers	0.2	5.1	1.8	0.5	2.4	2.3
Allowed	3.0	4.6	2.2	4.3	4.2	3.5
Favoured	96.8	90.3	96.0	95.2	93.4	94.2

† References associated with the PDB files used in this table: 1dsu, Narayana *et al.* (1994 ▶); 4cbn (refined with *phenix.refine*) and 4cbo (refined with *phenix.ensemble_refinement*), this work; 2xw9, Forneris *et al.* (2010 ▶); 1dst, Kim *et al.* (1995*a* ▶); 4d9r and 4d9q, Katschke *et al.* (2012 ▶).

‡ Values in parentheses are those reported in the original PDB entry.

§ For the two large FD–antibody structures determined at lower resolution, automatic default selection of τ_*x*_ yielded very short averaging windows; simulation of these structures were performed with τ_*x*_ set to 1.0 ps.

**Table 3:** Re-refinement and ensemble-refinement statistics for thrombin data sets

	WT, Na^+^-bound	PPACK Cys	PPACK Ile	PPACK Thr	S195A	WT, Na^+^-free	E217K, Na^+^-free	D102N, Na^+^-free
PDB code†	3u69	3u8t	3u8r	3u8o	1jou	2afq	1rd3	3bei
Resolution (Å)	1.6	1.9	1.5	1.3	1.8	1.9	2.5	1.5
*phenix.refine* re-refinement								
*R*_work_ ‡	0.147 (0.135)	0.141 (0.155)	0.143 (0.131)	0.145 (0.129)	0.155 (0.222)	0.148 (0.198)	0.191 (0.229)	0.156 (0.193)
*R*_free_ ‡	0.165 (0.167)	0.168 (0.195)	0.163 (0.161)	0.154 (0.149)	0.170 (0.245)	0.186 (0.229)	0.236 (0.259)	0.179 (0.215)
Geometric r.m.s.d.								
Bonds (Å)	0.009	0.008	0.010	0.008	0.012	0.012	0.003	0.020
Angles (°)	1.28	1.24	1.36	1.34	1.36	1.347	0.79	1.76
Dihedrals (°)	16.3	13.7	14.2	13.6	14.2	14.0	13.9	15.4
Ramachandran statistics (%)								
Outliers	0.0	0.0	0.0	0.0	0.8	0.7	0.9	1.4
Allowed	3.4	3.6	2.8	3.6	3.4	2.9	6.3	3.2
Favoured	96.6	96.4	97.2	96.4	95.9	96.3	92.8	95.4
*phenix.ensemble_refinement*								
τ_*x*_ (ps)	0.8	0.6	0.9	1.2	0.6	0.6	0.3	0.9
No. of models	134	67	129	127	89	100	60	77
*R*_work_	0.124	0.123	0.122	0.114	0.140	0.139	0.174	0.140
*R*_free_	0.155	0.161	0.148	0.135	0.167	0.174	0.218	0.163
Geometric r.m.s.d. (centroid distribution)								
Bonds (Å)	0.008	0.009	0.008	0.009	0.010	0.010	0.012	0.011
Angles (°)	1.15	1.21	1.18	1.28	1.12	1.12	1.10	1.12
Dihedrals (°)	8.4	9.0	8.2	8.5	8.2	8.4	9.1	8.1
Geometric r.m.s.d. (whole distribution)								
Bonds (Å)	0.015	0.016	0.016	0.017	0.016	0.016	0.016	0.018
Angles (°)	1.93	1.79	1.96	2.07	1.79	1.77	1.69	2.04
Dihedrals (°)	20.2	18.0	18.8	18.5	18.8	18.5	19.7	19.1
Ramachandran statistics (whole distribution) (%)								
Outliers	2.8	1.6	2.0	1.3	3.3	3.3	4.1	3.7
Allowed	6.5	6.3	5.8	6.0	6.7	6.2	10.9	6.7
Favoured	90.7	92.1	92.1	92.7	89.8	90.5	85.8	89.4
Ramachandran statistics (per torsion angle) (%)								
Outliers	1. 5	1.5	0.4	0.4	1.0	2.0	1.2	3.5
Allowed	3.6	4.4	3.3	3.6	2.1	2.4	3.1	2.2
Favoured	94.9	94.1	96.4	96.0	97.0	95.6	95.6	94.4

† References associated with the PDB files used in this table: 3u69, 3u8t, 3u8r and 3u8o, Figueiredo *et al.* (2012 ▶); 1jou, Huntington & Esmon (2003 ▶); 2afq, Johnson *et al.* (2005 ▶); 1rd3, Carter *et al.* (2004 ▶); 3bei, Gandhi *et al.* (2008 ▶).

‡ Values in parentheses are those reported in the original PDB entry.

## Footnotes

1 Supporting information has been deposited in the IUCr electronic archive (Reference: RR5061).
