# Supplementary material for: Ensemble refinement shows conformational flexibility in crystal structures of human complement factor D
Source: Acta Crystallogr D Biol Crystallogr. 2014 Feb 15;70(Pt 3):733–43. doi: 10.1107/S1399004713032549 (PMC3949522; doi:10.1107/S1399004713032549)
Supplement: Supplementary file 1 [file d-70-00733-sup1.pdf]

**Supplementary Information for**

# Ensemble refinement shows conformational flexibility in crystal structures of human complement factor D

**Federico Forneris[a,b], B. Tom Burnley[a,b,c] and Piet Gros[a]***

[a] Crystal and Structural Chemistry, Bijvoet Center for Biomolecular Research, Department of Chemistry, Faculty of Science, Utrecht University, Padualaan 8, Utrecht, 3584CH, The Netherlands

[b] These authors contributed equally

[c] Present Address: Scientific Computing Department, STFC, Research Complex at Harwell, Rutherford Appleton Laboratory, Didcot, Oxon OX11 0QX, UK.

* Correspondence email: p.gros@uu.nl, phone: +31-30-2533127, fax: +31-30-2533940

## List of Supplementary Figures:

- **Supplementary Figure S1** Structural features of complement Factor D and thrombin.
- **Supplementary Figure S2** Electron density for the catalytic site of FD R202A (R218A) mutant at 1.8-Å resolution.
- **Supplementary Figure S3** Improvement of electron density in disordered exosite regions after ER.
- **Supplementary Figure S4** Real-space cross-correlation for the 10 ensembles and B factor plots obtained from ER of FD and thrombin structures.
- **Supplementary Figure S5** Biophysical analysis of FD mutants.
- **Supplementary Figure S6** ER of crystal structures of FD in complex with an antibody fragment.

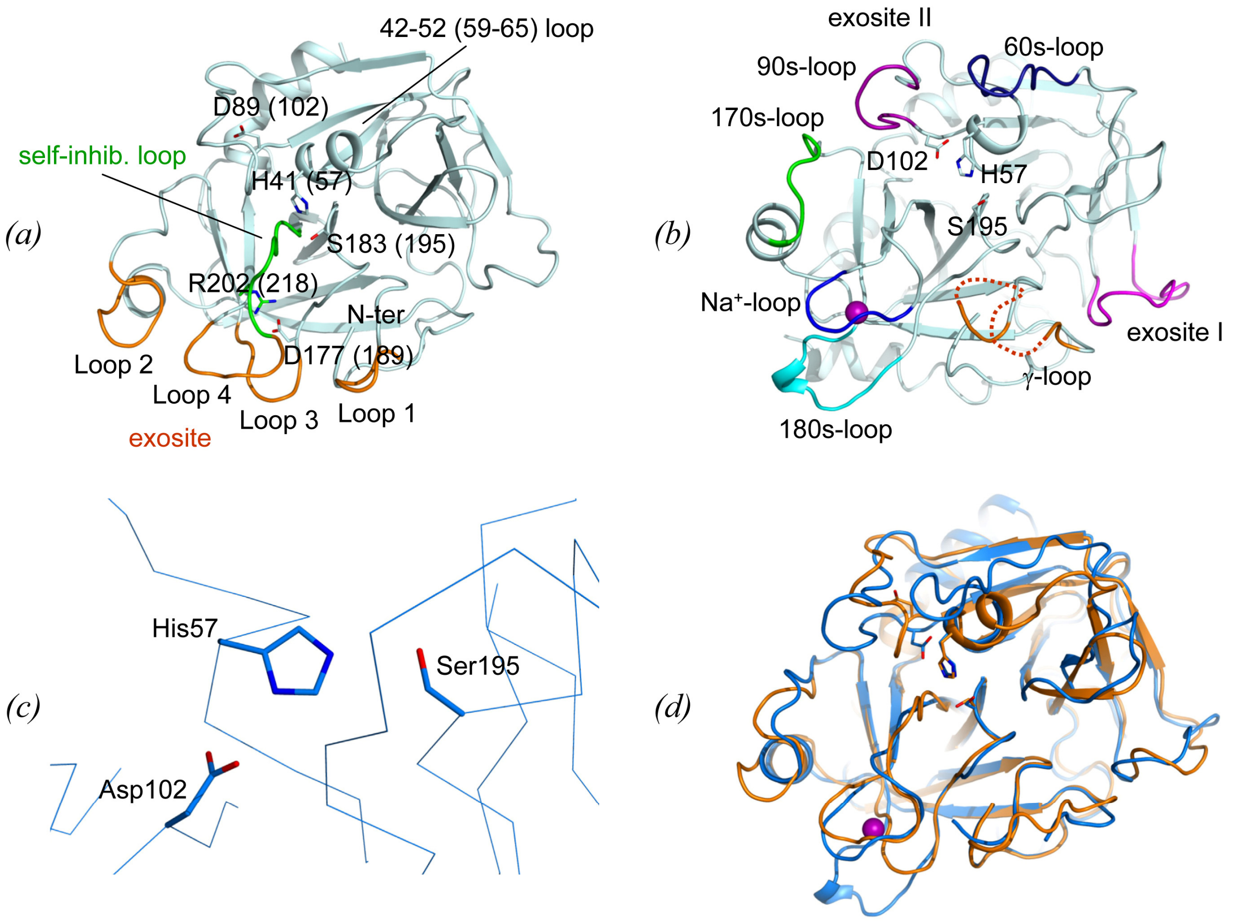


**Supplementary Figure S1** Structural features of complement Factor D and thrombin. *(a)* Cartoon representation of wild-type FD (PDB ID 1DSU, chain A is displayed). The side chains of the residues involved in the catalytic triad His-Asp-Ser, and those of the Arg202-Asp177 (Arg218-Asp189) self-inhibitory lock are shown as sticks. The four loops defining the exosite are highlighted in orange, and the self-inhibitory loop is shown in green. *(b)* Cartoon representation of wild-type, $Na^+$-bound thrombin (PDB ID 3U69). The side chains of residues of the catalytic triad are shown as sticks, with residue numbering following standard serine protease conventions. The sodium ion is shown as a purple sphere. Several loops involved in ligand binding and with implications in thrombin allosteric regulation are labeled and highlighted with colors. The missing residues of the γ-loop, not visible in this structure because of disorder, are represented by a dashed line. *(c)* Zoomed-in view of the catalytic site of wild-type thrombin, showing the canonical arrangement of the His-Asp-Ser catalytic triad. *(d)* Superposition of the FD (orange) and thrombin (blue) structures, showing the overlap of FD's exosite with the $Na^+$ binding site of thrombin. Residues of the catalytic triad are shown as sticks.

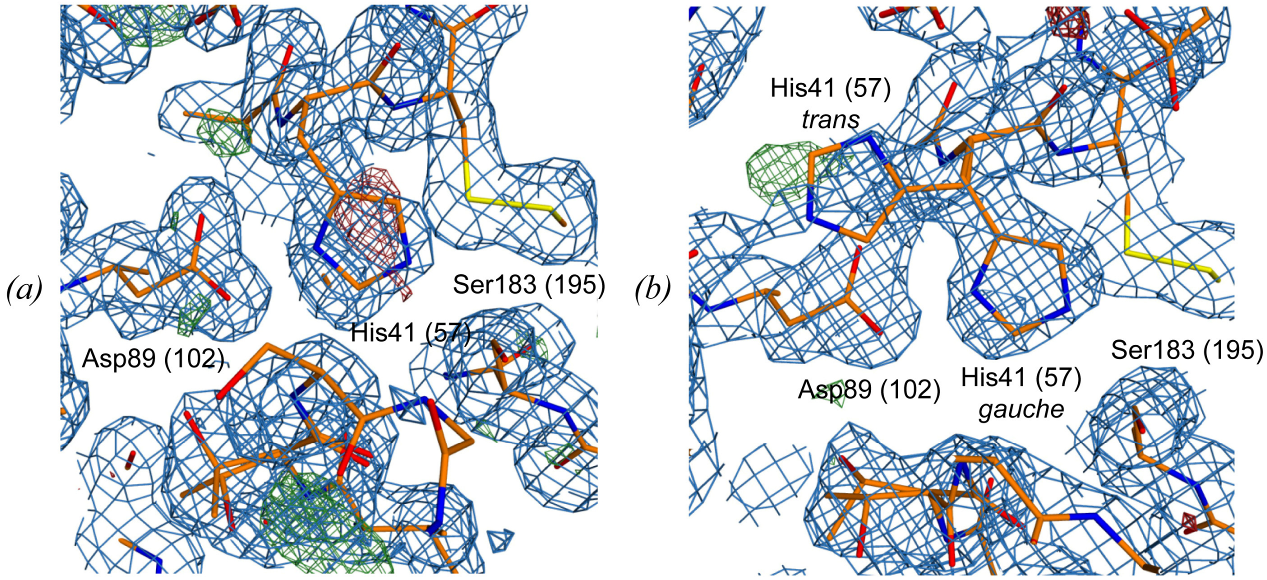


**Supplementary Figure S2** Refined electron density map for the Asp-His-Ser catalytic triad of FD R202A (R218A) mutant at 1.8-Å resolution after *phenix.refine* in the two copies observed in the asymmetric unit (shown as *(a)* and *(b)*, respectively). Residues are shown as orange sticks. The contour levels for the electron density maps are 1.00 σ ($2mF_o$-$DF_m$, blue) and ±3.00 σ ($mF_o$-$DF_m$, green and red).

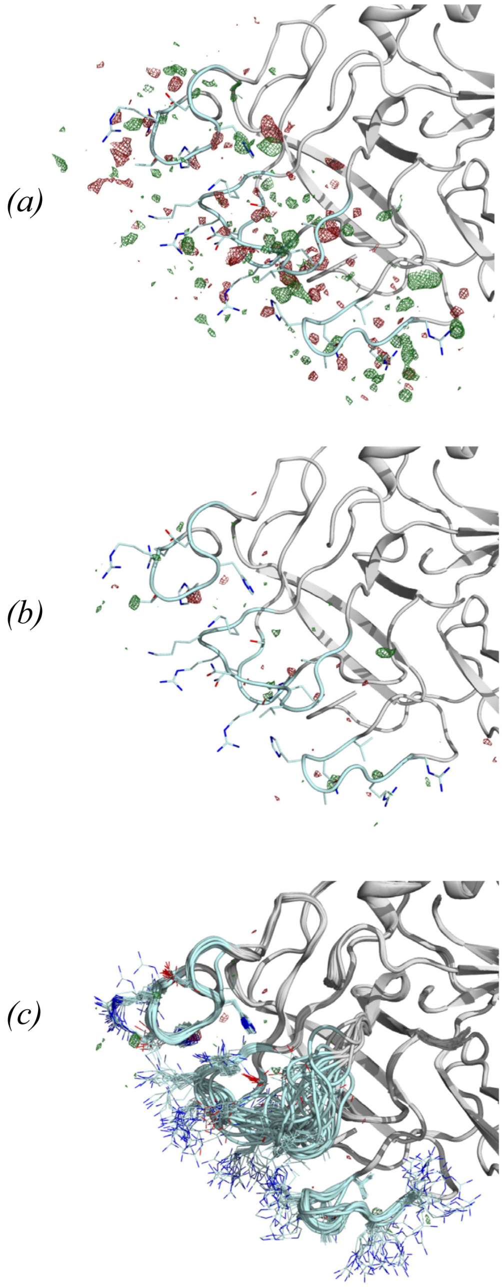


**Supplementary Figure S3** ER shows electron density improvement in disordered exosite regions. Comparison of electron densities of refined FD R202A (R218A) structures in the exosite region at equivalent electron density levels after conventional refinement and ER. *(a)* Conventional refinement structure and $mF_o$-$DF_m$ map contoured at ±0.31 Å/e$^3$ (±2.44 σ). *(b)* Conventional structure with ensemble map contoured at ±0.31 Å/e$^3$ (±3.00 σ). *(c)* Ensemble structure and ensemble map contoured as in *(b)*.

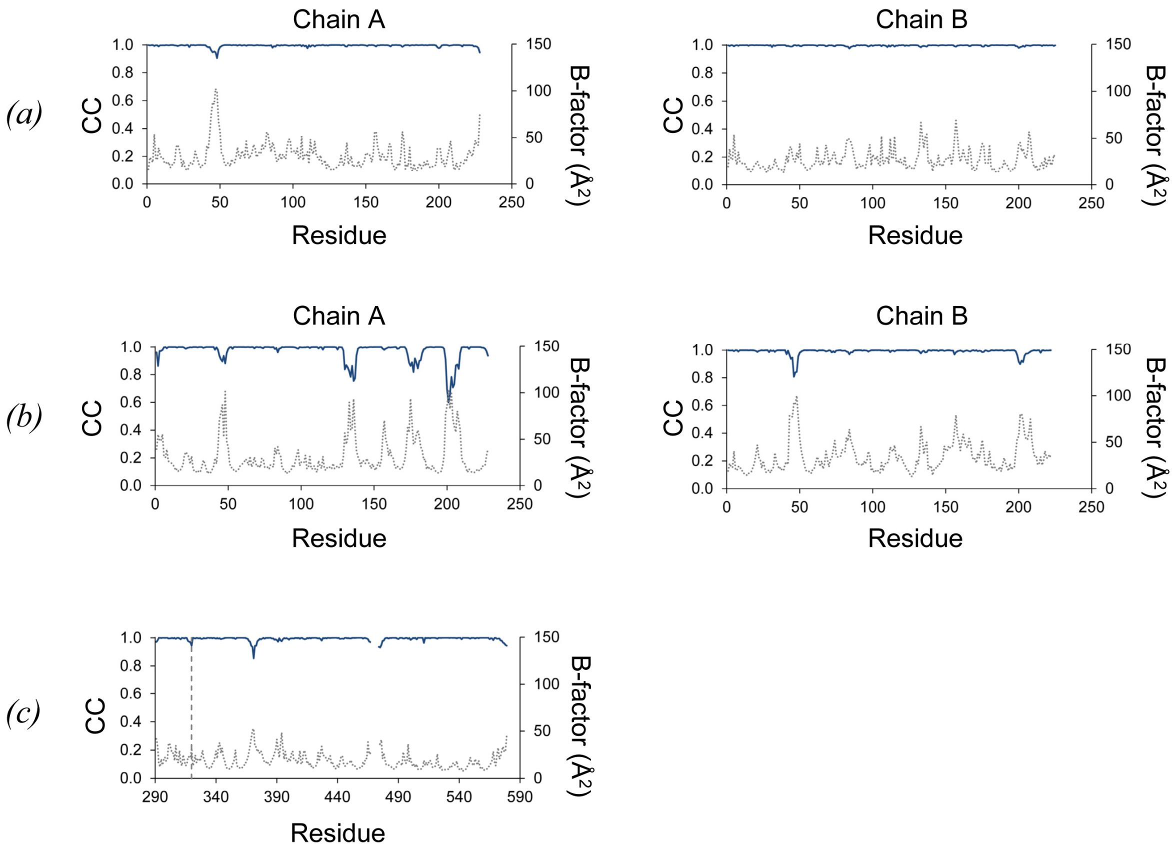


**Supplementary Figure S4** Real-space cross-correlation for the 10 ensembles (Branden & Alwyn Jones, 1990). In addition, B-factor as obtained from single-structure refinement of FD and thrombin structures are shown by dotted lines. *(a)* wild-type FD (original PDB ID 1DSU, 2 chains in the ASU). *(b)* FD mutant R202A (R128A) (original PDB ID 4CBN, 2 chains in the ASU). *(c)* wild-type, $Na^+$-bound thrombin (original PDB ID 3U69); the dashed gray line indicates the separation between thrombin light and heavy chains. Regions with low real-space correlation correspond to flexible loops as indicated by the high B-factors.

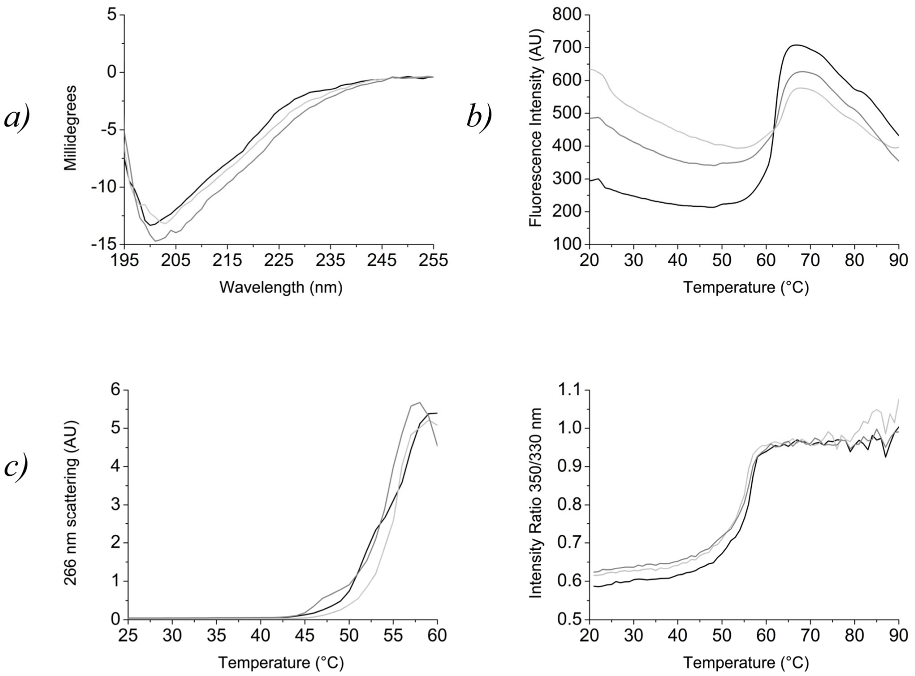


**Supplementary Figure S5** Biophysical analysis of FD mutants. *(a)* Circular dichroism measurements; *(b)* evaluation of thermal stability using thermofluor The calculated $T_m$ values are for FD wild-type, R202A and R106A mutants are 62.8 °C, 63.4 °C, and 63.8 °C, respectively; *(c)* measurement of thermal-dependent aggregation propensity (left) and unfolding monitored using intrinsic fluorescence (right). The calculated $T_m$ values are for FD wild-type, R202A (R218A) and R106A (R121A) mutants are 56.5 °C, 54.9 °C, and 55.0 °C, respectively. Shown are traces for wild-type FD (black), mutant R202A (R218A) (dark grey) and control mutant R106A (R121A) (light grey).

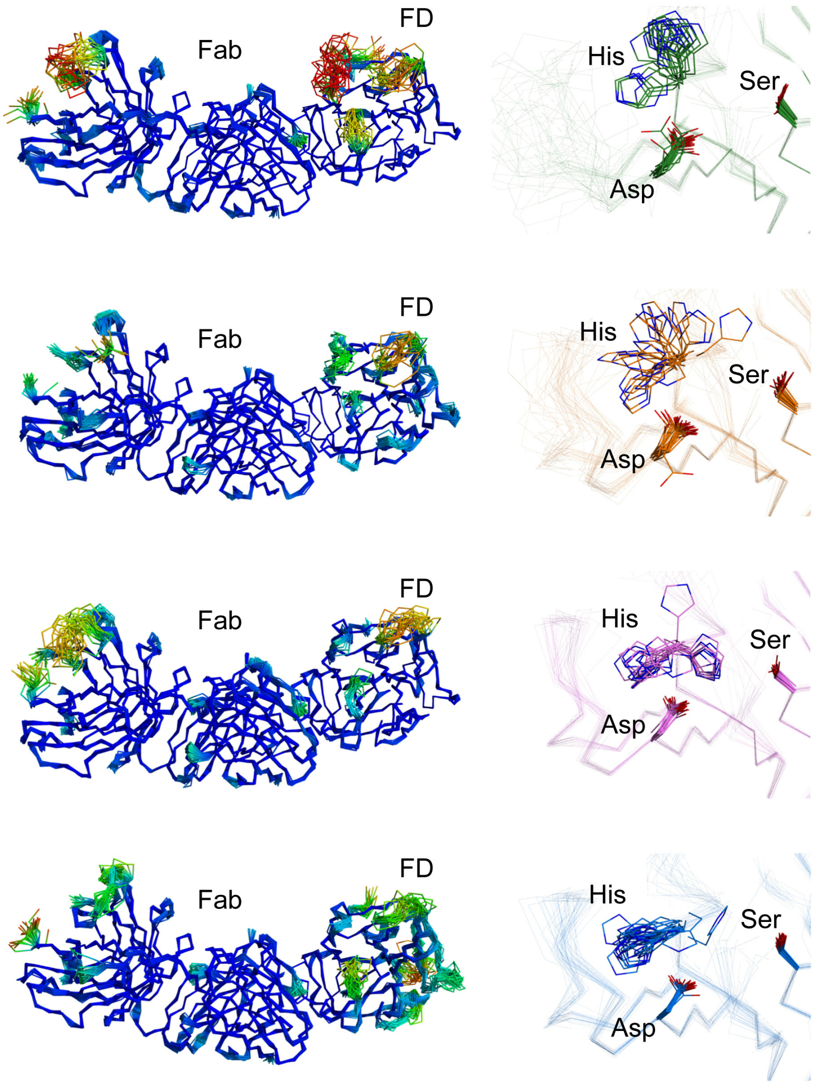


**Supplementary Figure S6** ER of crystal structures of FD in complex with an antibody fragment. From top to bottom, the two copies of the FD-Fab complex observed in each structure originally deposited in the PDB with accession codes 4D9Q (top) and 4D9R (bottom) (Katschke *et al.*, 2012) are displayed. On the left, the overall FD-Fab complexes are represented as ribbons, with models colored by rmsf from blue, through green, to red. On the right, details are shown for the Asp-His-Ser catalytic triad in each copy of the FD molecule.
